# Knowledge, attitude and practice of hygiene and sanitation in a Burundian refugee camp: implications for control of a Salmonella typhi outbreak

**DOI:** 10.11604/pamj.2017.28.54.12265

**Published:** 2017-09-21

**Authors:** Marie-Rosette Nahimana, Candide Tran Ngoc, Olushayo Olu, Jose Nyamusore, Ayodeji Isiaka, Vedaste Ndahindwa, Lakruwan Dassanayake, André Rusanganwa

**Affiliations:** 1 WHO Country Office, Ebenezer House, Boulevard of Umuganda, Kigali, Rwanda; 2 Rwanda Biomedical Center, Kigali, Rwanda; 3 School of Public Health, Kigali, Rwanda; 4 UNHCR, Kigali, Rwanda

**Keywords:** Water, sanitation, hygiene, knowledge, attitude, practice, Burundian refugees, Salmonella typhi outbreak

## Abstract

**Introduction:**

A *Salmonella typhi* outbreak was reported in a Burundian refugee camp in Rwanda in October 2015. Transmission persisted despite increased hygiene promotion activities and hand-washing facilities instituted to prevent and control the outbreak. A knowledge, attitude and practice (KAP) study was carried out to assess the effectiveness of ongoing typhoid fever preventive interventions.

**Methods:**

A cross-sectional survey was conducted in Mahama Refugee Camp of Kirehe District, Rwanda from January to February 2016. Data were obtained through administration of a structured KAP questionnaire. Descriptive, bivariate and multivariate analysis was performed using STATA software.

**Results:**

A total of 671 respondents comprising 264 (39.3%) males and 407 (60.7%) females were enrolled in the study. A comparison of hand washing practices before and after institution of prevention and control measures showed a 37% increase in the proportion of respondents who washed their hands before eating and after using the toilet (p < 0.001). About 52.8% of participants reported having heard about typhoid fever, however 25.9% had received health education. Only 34.6% and 38.6% of the respondents respectively knew how typhoid fever spreads and is prevented. Most respondents (98.2%) used pit latrines for disposal of feces. Long duration of stay in the camp, age over 35 years and being unemployed were statistically associated with poor hand washing practices.

**Conclusion:**

The findings of this study underline the need for bolstering up health education and hygiene promotion activities in Mahama and other refugee camp settings.

## Introduction

Disputes over political and religious ideology, natural resources and ethnic identity are common causes of armed and civil conflicts, which in turn result in population displacement. In 2014, 33% (19.5 million) of the total number of people displaced globally (59.5 million) were refugees, of which 19% lived in Sub-Saharan Africa [1]. In May 2015, a major political crisis in Burundi culminated in street demonstrations and violent confrontations between a government-backed militia and local populations. As a result, many Burundians fled across the border into neighboring countries such as Tanzania and Rwanda. As of March 2016, the number of Burundian refugees registered in Rwanda reached 76,127, of which 47,908 were accommodated in a refugee camp in the Mahama sector of Kirehe District in Eastern Province of Rwanda [2]. Beginning in late October 2015, health facilities in the camp recorded increased incidence of a febrile illness among inhabitants of the camp. The Ministry of Health (MOH), in collaboration with the Ministry of Disaster Management and Refugee Affairs (MIDIMAR) and other partners working in the camp, conducted an investigation and on 1 December 2015, confirmed an outbreak of typhoid fever. As of the end of January 2016, an estimated 1663 cases had been reported. The causative organism of typhoid fever is the bacterium Salmonella enterica serotype Typhi, which is transmitted through ingestion of food and water contaminated by the feces and urine of patients or asymptomatic carriers of the bacteria [3]. Clinical symptoms of the disease are relatively mild and include fever, abdominal pain, malaise, nausea (and sometimes vomiting) and loss of appetite [4]. However, the clinical scope may vary from mild illness with low-grade fever to severe clinical disease with abdominal discomfort and intestinal perforation [4, 5].

Without effective treatment, the case fatality rate is 10% to 30% [6]. Interventions for disease prevention include hygiene education on and practice of safe handling of food [7], hand washing with soap and drinking safe water [8]. The United Nations High Commissioner for Refugees (UNHCR) together with relevant sectors of the Government of Rwanda and its partners, including the World Health Organization (WHO), immediately instituted outbreak prevention and control measures. These measures included improvements in the quantity and quality of the water supply through water quality testing and chlorination of drinking water, hygiene education and community mobilization on safe food handling and waste disposal, improved sanitation through provision of more latrine stands, active detection and appropriate management of cases. Despite these interventions, the outbreak situation continued to deteriorate, with an average of 26 new suspected cases reported per day from 7 December 2015 to 8 January 2016. This prompted the need to better understand the risk factors for disease transmission and determine the impact of preventive and control measures. A knowledge, attitude and practice (KAP) study toward typhoid fever was therefore conducted. The KAP survey of Mahama camp residents sought to determine whether hygiene and sanitation as they relate to typhoid fever prevention was adequate. The objectives of the study were to assess the effectiveness of ongoing typhoid fever preventive interventions; the results were expected to be used to review the outbreak intervention strategies and institute immediate actions.

## Methods

**Study design**: A hygiene and sanitation KAP study was conducted from January to February 2016, as part of a case-control study to identify risk factors for transmission of Salmonella typhi in Mahama Refugee Camp of Kirehe District, Rwanda. Data for the study were obtained from a cross-sectional survey of residents of the camp using a structured KAP questionnaire and observation of sanitation and hygiene practices.

**Study area**: Mahama camp is located in the South-eastern part of Rwanda and was established in April 2015 to house Burundian refugees (Annex 1 for map of camp). The camp has a surface area of 50 hectares and accommodates 18,360 households, distributed among 15 villages at two main sites, namely, the old and new sites of the camp. Residents in the old site were housed in temporary shelters (tents) whereas those in the new site lived in semi-permanent structures. Water was supplied to the camp via a mini water treatment plant, which draws its supply from the nearby Akagera River. Sanitation facilities were provided through improved pit latrines; each latrine drop hole was shared by an average of 20 to 25 persons. Hand washing facilities were provided at each latrine block. As of January 2016, the quantity of water supplied had reached 15-20 liters per person per day in both sites, which was above the minimum standard required for refugee camps [9]. The camp had two health centers that deliver both treatment and preventive care services, including community mobilization, health and hygiene education. According to health center data, the top three causes of morbidity were respiratory tract infections, malaria and watery diarrhea.

**Sample size and sampling methods**: The sample size for the original case control study was generated using a method described by Lemeshow et al [10], which estimated the sample size at 1,036 camp residents (259 cases and 777 controls). For each case, three age and sex matched controls were selected. An additional file shows the sample size calculation in more details (Annex 2). A systematic random sampling method was used to select cases from a list provided by the camp health center. Each interviewer was given a list of respondents (cases) and interviewers worked with village leaders and community health workers (CHWs) to identify households of cases. A systematic random sampling method was then used to identify the controls for each case. After each case was interviewed, the interviewer skipped the two households to the right of the case's house and selected controls from the next (third) household who were of the same sex and age group as the case. The same was done for the left side of the case household, until the required number of controls for that case had been selected and interviewed. In the event that the household chosen had no eligible candidate or a family member was a case, the surveyor moved to the next household.

**Data collection**: Data for this study were obtained through administration of a structured KAP questionnaire to both cases and controls who had participated in the case-control study. The questionnaire consisted of 32 questions that were categorized into four main sections: sociodemographic characteristics, knowledge, attitude and practice (Annex 3 for the questionnaire). Some of the questions on attitude and practice were also asked retrospectively to explore adherence before and after institution of control actions. The questionnaire mainly comprised close-ended questions that were developed in English and translated into Kinyarwanda, a language well understood by both Burundians and Rwandans. Interviewers provided a list of options to the interviewees and then marked the most appropriate based on the response. Knowledge-related questions addressed knowledge about typhoid fever and how it is transmitted and prevented; attitude questions included the health-seeking behaviors of respondents. Practice-related questions queried the use of fecal disposal facilities and hand washing practices, including frequency of hand washing before eating and after using the toilet, for the periods prior to December 2015 and after the institution of preventive and control measures. Survey team was composed of fifteen experienced Rwandan nurses and laboratory technicians who were selected from neighboring government health facilities. They were trained on the survey and data collection methods. Prior to data collection, the interviewers pre-tested the questionnaire. Based on feedback from the pretest, the study team rephrased and rechecked question accuracy and adequacy and estimated the time needed to conduct the interviews. To validate the findings of the quantitative survey and identify practical issues around sanitation and hygiene behavior, a team comprising two study team members and an environmental health expert conducted environmental assessments. During these assessments, observation of participants' hygiene practices was carried out. The assessment team conducted several transect walks through the camp, randomly visiting and observing activities at the mini-water treatment plant, water distribution points, hand-washing stations, households, camp markets and health centers. At each station, the team observed and recorded various aspects of hygiene practices, especially hand washing.

**Data analysis**: A database was established in the Census and Survey Processing System (CSPro) version 6.1 [11] and data were entered directly by interviewers using Internet-enabled tablet computers. Data were exported into Stata 13 software [12] and cleaned. Two exclusion criteria were applied to the database; 1) any person who had not resided in the camp for at least one month prior to the study; these category of persons were excluded due to their short and inadequate exposure to the preventive interventions which were being assessed in this study and 2) any person below the age of 15, to exclude responses provided by caregivers. Descriptive, bivariate and multivariable analysis were performed on the subset of respondents who met the inclusion criteria. Descriptive analyses included frequencies of socio-demographic characteristics such as age, sex, education etc, knowledge, attitude and practice of hygiene and sanitation. We also compared the response of cases and controls, to assess whether there was any variation. To determine the association between socio-demographic characteristics and respondents' KAP, we conducted bivariate and multivariable analyses, which cross-tabulated time spent in the camp, age, education level, occupation and religion with KAP variables of interest such as hand washing, method of fecal disposal, knowledge of typhoid fever and health education. We applied a backward variable selection method in logistic regression and calculated adjusted odds ratios (AOR), 95% confidence intervals (CI) and p-values, to determine which socio-demographic factors were independently associated with low KAP about typhoid fever prevention. A threshold of 0.05 was used for statistical significance.

**Ethics consideration**: Ethical clearance for the study was given by the Rwanda National Ethical Committee. Before conducting the interviews, informed consent was obtained from all study participants after an explanation of the purpose of the study. Approval to conduct the study and publish its results was obtained from the WHO (e-Pub number ePub-IP-00043431-EC), MIDIMAR and UNHCR Rwanda.

## Results

**Socio-demographic characteristics**: A total 671 respondents comprising 264 (39.3%) males and 407 (60.7%) females were enrolled into the study; controls and cases accounted for respectively 74.7% and 25.3% of participants. Five hundred and two (74.8%) respondents had resided in the camp for more than 6 months. Most respondents were aged 25-34 years (36.1%) followed by 15-24 years (32.9%). Two hundred and twelve (31.6%) respondents had no formal education; however, 41.7% and 23.7%, respectively, had completed primary and secondary education. More than half (57.8%) were unemployed; 48.7% and 36.8%, respectively, were Catholic and Protestant (Table 1).

**Table 1:** Socio-demographic characteristics of respondents in Mahama camp, Rwanda (2016)

Variable	Category	Number	%
Age (years)	15–24	221	32.9
	25–34	242	36.1
	35–44	90	13.4
	45–54	55	8.2
	≥55	63	9.4
Sex	Male	264	39.3
	Female	407	60.7
Time spent in the camp	1–6 months	169	25.2
	More than 6 months	502	74.8
Educational level	None	212	31.6
	Pre-school	10	1.5
	Primary	280	41.7
	Secondary	159	23.7
	Tertiary	10	1.5
Religion	None	28	4.2
	Catholic	326	48.6
	Protestant	247	36.8
	Adventist	25	3.7
	Muslim	45	6.7
Occupation	None	388	57.8
	Farming	103	15.4
	Trading	28	4.2
	Caterer	53	7.9
	Student	99	14.8
Marital status	Single	234	34.87
	Currently in union	354	52.76
	Separated/Divorced/Widowed	83	12.37

**Knowledge and attitude of hygiene and sanitation**: Three hundred and fifty four (52.8%) participants reported to have heard about typhoid fever before the outbreak. The MOH was the most common source of information reported by respondents (50.3%) followed by community meetings (31.6%). Only 26% of respondents reported having received health education during the course of the outbreak. Among this group, sources of health education reported were health facility staff (44.8%), house-to-house hygiene promoters (46.5%) and community meetings (45.4%). The level of knowledge about how typhoid fever is transmitted and prevented was low among study participants; only 34.6% and 38.6%, respectively, knew how typhoid fever spreads and how it is prevented. Many respondents (82.3%) identified eating contaminated food as the manner in which typhoid fever infection is contracted, followed by eating with dirty hands (75%) and drinking contaminated water (66.8%). The most commonly mentioned methods of preventing typhoid fever were washing hands with soap before eating (87.3%), drinking boiled water (75.7%) and washing hands before cooking, cooking food thoroughly and eating food while hot(75.7%). Most respondents (96%) reported seeking medical assistance when sick, with 95.8% of them seeking care from orthodox sources of health care (Table 2).

**Table 2:** Knowledge and attitude of *Salmonella* typhi prevention and control methods in Mahama camp, Rwanda (2016)

Variables	**Responses**	**Number**	**%**	**95% CI**
Heard about typhoid fever	Yes	354	52.8	49.0–56.5
	No	317	47.2	43.5–51.0
Source of information on typhoid fever	Ministry of Health/health workers	178	50.3	45.1–55.5
	Media (TV, radio, newspapers, posters…)	79	22.3	18.3–27.0
	Community meeting	112	31.6	27.0–36.7
Received health education	Yes	174	26	22.7–29.4
	No	497	74	71.6–77.3
Source of health education	Heath facility staff	78	44.8	37.5–52.3
	House-to-house hygiene promoters	79	45.4	38.1–52.9
	Street campaigns	17	9.8	6.1–15.2
	Place of worship	4	2.3	0.85–6.0
	Camp school	12	6.9	3.9–11.8
	Community meetings	81	46.5	37.9–54.0
Knowledge of how typhoid fever spreads	Yes	232	34.6	31.0–38.2
	No	439	65.4	61.7–68.9
Knowledge of how typhoid fever is transmitted	Drinking contaminated water	155	66.8	60.4–72.6
	Eating contaminated food	191	82.3	76.8–86.7
	Eating with dirty hands	174	75	69.0–80.2
	Contact between houseflies and food	87	37.5	31.4–43.9
	Contact with vomit or stool	59	25.4	20.2–31.5
	Drinking water stored over one day	43	18.5	14.0–24.1
Knowledge of how typhoid fever is prevented	Yes	259	38.6	35.0–42.3
	No	412	61.4	57.6–65.0
Knowledge of ways to prevent typhoid fever	Drinking treated and boiled water	196	75.7	70.0–80.5
	Wash hands with soap before eating and after leaving the toilet	226	87.3	62.6–90.8
	Washing hands before cooking , cooking food thoroughly and eating food while hot	196	75.7	70.0–80.5
	Washing fruit, vegetables and boiling vegetables before eating	137	52.9	46.8–58.9

**Practice of hygiene methods for prevention and control of *Salmonella* typhi**: When asked question on what fecal disposal facilities they frequently use, most respondents (98.2%) reported using pit latrines for feces disposal. Respectively 61.5% and 59.0% of respondents reported that they always washed their hands before eating and after using the latrine prior to the time that preventive and control measures were instated; during the same period, close to 25% reported that they sometimes or never washed their hands. At the time of data collection, the proportion of people who reported to always wash their hands before eating and after using the latrine had increased significantly to 71.3% and 71.7%, respectively (p < 0.001). About 87.0% of respondents reported using soap when they wash their hands and 13% used water only. With respect to knowledge, attitudes and practices, no significant differences were observed between cases and controls (Table 3).

**Table 3:** Distribution of KAP about *Salmonella* typhi prevention among controls and cases in Mahama camp, Rwanda (2016)

KAP	Responses	Case (%)	Controls (%)	Total (%)
Washed hands before eating prior to December 2015	Always	125 (73.5)	388 (77.5)	513 (76.5)
	Sometimes or never	45 (26.5)	113 (22.5)	158 (23.5)
Washed hands after toilet prior to December 2015	Always	122 (71.8)	384 (76.7)	506 (75.4)
	Sometimes or never	48 (28.2)	117 (23.3)	165 (24.6)
Currently washes hands before eating	Always	163 (96.5)	474 (95.9)	637 (96.1)
	Most of the time	6 (3.5)	20 (4.1)	26 (3.9)
Currently washes hands after toilet	Always	164 (97.0)	471 (95.5)	635 (95.9)
	Sometimes or never	5 (3.0)	22 (4.5)	27 (4.1)
Has heard about typhoid fever	Yes	82 (48.2)	272 (54.3)	354 (52.8)
	No	88 (51.7)	229 (45.7)	317 (47.2)
Received health education	Yes	41 (24.1)	133 (26.50	174 (26.0)
	No	129 (75.9)	368 (73.5)	497 (74.0)
Knows how typhoid fever spreads	Yes	57 (33.5)	175 (34.9)	232 (34.6)
	No	113 (66.5)	326 (65.1)	439 (65.4)
Knows how typhoid fever is prevented	Yes	64 (37.5)	195 (38.9)	259 (38.6)
	No	106 (62.3)	306 (61.1)	412 (61.4)
Seeks medical assistance when sick	Yes	160 (94.1)	484 (96.6)	644 (96.0)
	No	10 (5.9)	17 (3.4)	27 (4.0)

**Association between education level, occupation, religion, age and level of knowledge, attitude and practice**: Respondents who had spent more than 6 months in the camp (OR 1.86, p = 0.008) and those aged over 35 years were less likely to wash their hands before eating and after using the latrine. However, study participants who were employed were more likely to wash their hands (OR 0.49, p = 0.001). Concerning knowledge and information about typhoid fever, respondents who had resided in the camp for more than 6 months were less likely to have heard about typhoid fever (OR 2.31, p = 0.000) and to know how the disease is transmitted (OR 1.80, p = 0.002) or prevented (OR 1.76, p = 0.002). However, respondents aged over 35 years were more likely to have received health education (OR 0.64, p = 0.016) about typhoid fever. With respect to education and religion, respondents who had completed primary and secondary level of education and those who were protestant were more likely to have heard about typhoid fever and to know how it spreads and is prevented (Table 4). An additional file shows the full results of the bivariate and multivariate analysis (Annex 4).

**Table 4:** Association between selected socio-demographic characteristics and level of KAP in Mahama refugee camp, Rwanda (2016)

KAP variables	Socio-demographic characteristics	%	UOR (95% CI)	p-value	AOR (95% CI)	P-value
Does not wash hands before eating	> 6 months in the camp	26.1	1.86 (1.17–2.93)	0.008	1.86 (1.17–2.96)	0.008
	Age ≥ 35 years	28.9	1.51 (1.04–2.2)	0.031	1.47 (1.01–2.16)	0.045
	Employed	16.3	0.48 (0.32–0.70)	<0.001	0.49 (0.33–0.73)	<0.001
Does not wash hands after using latrine	> 6 months in the camp	27.9	2.23 (1.39–3.55)	<0.001	2.24 (1.40–3.61)	0.001
	Age ≥ 35 years	29.8	1.48 (1.03–2.15)	0.036	1.47 (1.05–2.32)	0.047
	Employed	17.3	0.49 (0.33–071)	<0.001	0.50 (0.34–0.71)	0.001
Fecal disposal other than pit latrine	> 6 months in the camp	0.6	0.10 (0.30–0.40)	0.001	0.10 (0.02–0.36)	0.001
	Employed	0.4	0.12 (0.02–0.94)	0.044	0.10 (0.01–0.81)	0.033
Has not heard about typhoid fever	> 6 months in the camp	52.4	2.34 (1.62–3.38)	<0.001	2.31 (1.59–3.35)	<0.001
	Primary school and high	43.2	0.61 (0.44–0.84)	0.003	0.64 (0.46–0.90)	0.003
	Protestant	43.7	0.51 (0.30–0.87)	0.013	0.52 (0.30–0.89)	0.018
Has not received health education	Age ≥ 35 years	67.8	0.63 (0.44–0.91)	0.013	0.64 (0.44–0.92)	0.016
Does not know how typhoid fever spreads	> 6 months in the camp	68.9	1.81 (1.27–2.59)	0.001	1.80 (1.25–2.58)	0.002
	Primary school and high	60.4	0.49 (0.34–0.70)	<0.001	0.50 (0.34–0.72)	<0.001
	Protestant	61.4	0.52 (0.59–0.96)	0.029	0.52 (0.29–0.95)	0.034
Does not know how typhoid fever is prevented	> 6 months in the camp	64.9	1.79 (1.25–2.54)	0.001	1.76 (1.22–2.52)	0.002
	Primary school and high	56.6	0.53 (0.37–0.74)	<0.001	0.54 (0.37–0.77)	0.001
	Catholic	62.0	0.49 (0.27–0.89)	0.019	0.53(0.29–0.96)	0.037
	Protestant	56.6	0.40 (0.22–0.72)	0.002	0.40 (0.21–0.72)	0.003

**Observation of hygiene practices**: The observations revealed that most participants did not follow correct hand washing practices as prescribed by WHO. Other observed practices included the erecting of many temporary structures (tents) close to pit latrines, preparation of food close to waste water drainage ditches, use of open waste water ditches as play areas for children and placement of pipes carrying clean water inside waste water drainage ditches, among others.

## Discussion

Outbreaks of communicable diseases such as typhoid fever significantly contribute to increased morbidity and mortality during situations of mass population displacement. Poor environmental and living conditions, overcrowding and inadequate access to health and social services such as health care, clean water and adequate sanitation usually characterize such situations. These conditions increase the risk of outbreaks of water and foodborne diseases and hamper the timely control of those outbreaks. Hence, it is important to institute effective, evidence-based and participatory preventive and control measures to mitigate outbreaks as quickly as possible [13, 14]. Availability of adequate quantities of safe water, improved sanitation facilities, as well as very good knowledge, attitude and most importantly, consistent and correct practice of appropriate hygiene and sanitation methods are critical to the prevention and control of such outbreaks. Thorough understanding of these dynamics in refugee camps such as Mahama is therefore critical and this study contributes to that objective. In general, our findings showed that knowledge and attitude towards typhoid fever prevention and control was low among the study population, which may have contributed to the prolonged transmission of *Salmonella* typhi in the camp. Only a quarter of respondents had received health education about typhoid fever prevention and control during the outbreak and only 34% knew how the disease is transmitted despite ongoing community mobilization and hygiene education at the time of the study. Furthermore, less than 40% of respondents knew how typhoid fever can be prevented. House-to-house hygiene promotion, the main source of health education in the camp, accounted for less than 50% of the information provided to respondents; this demonstrates a further challenge to community mobilization and participation in hygiene education interventions in the camp.

This trend may be attributable to four main reasons. First, the coverage of community mobilization and hygiene education may have been inadequate to ensure sufficient knowledge levels and behavioral change among the camp population. Second, the methods of community mobilization and hygiene education may have been inappropriate for this setting. Experience has shown that in such settings, constant repetition and reinforcement of information is required to achieve behavioral change [15]. To this effect Participatory Hygiene and Sanitation Transformation (PHAST) approach could have been used as an incremental method to achieve required community participation in promoting hygiene and health [16]. Third, there may have been challenges to the quality of hygiene education messages and products used for community mobilization in the camp. Fourth, community hygiene promoters and Community Health Workers (CHWs) who were responsible for hygiene education about typhoid prevention and control may not have been fully trained in hygiene promotion and prevention of hygiene-related diseases. Thus, their inadequate knowledge and skills may have been a barrier to conducting adequate community mobilization and achieving quality hygiene education. Among study participants, there was low incidence of open defecation in the camp and improvement in the practice of typhoid fever prevention and control methods, such as hand washing. Between December 2015 and the time of the study, a 37% increase was seen in the proportion of respondents who washed their hands before eating and a 39% increase in the proportion who washed their hands after using the latrine. Furthermore, the proportion of respondents who reported to always wash their hands was high during the same period and 87% reported using soap for hand washing. These findings may be attributed to a sufficient number of provided latrines and hand washing stations located strategically in the camp. However, our observations of hygiene practices showed deficiencies in hand washing methods, which may be another reason for the prolonged outbreak. We observed that the hand washing methods used fell short of WHO recommendations [17]; thus, infective organisms such as typhoid bacteria may not be completely eliminated from camp residents' hands after using the latrine [18–20]. Given that the anal cleansing method is widely used for self-cleaning after latrine use, the risk of sustained transmission of typhoid bacteria is high [21].

Poor hand-washing practices might be linked to gaps in the health information provided to the community by the hygiene promoters and CHWs responsible for hygiene promotion and community mobilization. Our observation of practice also revealed several other high-risk behaviors, such as preparation and serving of food near open drainage systems, use of open drainage ditches as play areas for young children and poor management of waste water; an example of the latter is channeling waste water into ditches where pipes for clean water are laid, which may result in contamination through seepage. The outcomes of bi- and multivariate analysis showed that, in general, participants aged less than 35 years, those who were employed and those with some form of education (primary level or higher) were more likely to have better KAP of typhoid fever prevention and control methods. There is some evidence that people who spent longer time in a refugee camp are expected to have been exposed to hygiene promotion activities and would have adopted good hygiene practices [22], but this was not always the case in refugee's camps [23, 24]; similar findings were observed in Mahama camp. We hypothesize that those who had spent longer periods in the camp had become complacent, as compared with new arrivals. These findings are consistent with those of similar studies demonstrating an association between poor knowledge of foodborne diseases and socio-demographic characteristics, such as low education level and age under 45 years, which influenced the attitudes of food handlers at a medical college in India [25]. Our results have been further confirmed by studies conducted in Bangladesh, Nigeria, Tanzania, Cameroon, South Africa and South Sudan [23, 26–30]. These findings provided evidence for better targeting of community mobilization and hygiene education interventions. However, our findings were in contrast with those of similar studies in in Ghana and Pakistan [30, 31], which reported good knowledge, attitudes and practices towards typhoid fever in the surveyed communities.

**Study limitations**: First, this study was conducted 3 months after the occurrence of a typhoid fever outbreak, once measures for prevention and control of the outbreak, including hygiene promotion and health education, had been put in place; the KAP may have been altered by these ongoing interventions. This situation may have contributed to masking of some study results. We addressed this limitation by combining quantitative data collection with observation of hygiene practices. Second, the study data were retrospectively collected and self-reported, which may have introduced some elements of recall bias. Third, interviewee bias could have been a limitation as respondents may have answered questions based on their preconceived perception of the correct response. Fourth, we did not test the validity and reliability of the questionnaire. These limitations were mitigated through the rigorous training of data collectors and pretesting of the study questionnaire.

## Conclusion

This study confirmed low knowledge and attitude levels but good practice levels of typhoid fever prevention and control activities in the Mahama Burundian refugee's camp. However, practice methods of key hygiene activities, such as hand washing, were observed to be inadequate, which may be a plausible reason for the prolonged transmission of Salmonella Typhi in the camp. These findings highlight the fact that outbreak response interventions in the study population, particularly community mobilization and hygiene education, were ineffective. Based on these findings, we recommend reinforcement of health education and hygiene promotion activities in Mahama and other refugee camp settings. During future outbreaks, specific groups, such as people with low levels of education, those who are unemployed and youths should be targeted to receive appropriate hygiene, sanitation and health information. In addition to provision of water and soap, the promotion of the correct method and frequency of hand washing with soap and water is also recommended in this and other refugee camp situations. Furthermore, we recommend proper training, supervision and monitoring of community hygiene educators and CHWs. These recommendations were implemented in Mahama refugee camp by MIDIMAR, WHO, UNHCR and their implementing partners, resulting in a substantial decrease in the number of reported typhoid fever cases.

### What is known about this topic

- The bacterium *Salmonella enterica* serotype typhi, is transmitted through ingestion of food and water contaminated by the feces and urine of patients or asymptomatic carriers;
- Situations of mass population displacement such as refugee camps are fertile grounds for transmission of communicable diseases such as typhoid fever;
- Availability of adequate quantities of safe water, improved sanitation facilities, as well as very good knowledge, attitude and most importantly, consistent and correct practice of appropriate hygiene and sanitation methods are critical to the prevention and control of salmonella outbreaks.

### What this study adds

- Prevention and control of salmonella is not only linked to the level of practice of personal hygiene and sanitation interventions in camp setting but importantly to the correctness and consistency in the practice.

## Competing interests

The authors declare no competing interest.

## Annexes

**Annex 1**: map of Mahama camp, Rwanda

**Annex 2**: sample size calculation method

**Annex 3**: KAP survey questionnaire

**Annex 4**: Socio-demographic characteristics associated with KAP towards Typhoid fever prevention in Mahama refugee camp, 2016
